# The Launch of the Advanced Practitioner Society for Hematology and Oncology (APSHO)

**DOI:** 10.6004/jadpro.2014.5.2.1

**Published:** 2014-03-01

**Authors:** Pamela Hallquist Viale, Christopher J. Campen, Sandra E. Kurtin, Wendy H. Vogel

## Report from JADPRO Live

We are pleased to announce the formation of our new organization, the Advanced Practitioner Society for Hematology and Oncology (APSHO). The Journal of the Advanced Practitioner in Oncology (JADPRO) will be the Society’s official journal and will continue to offer relevant and high-quality articles focusing on the needs of the advanced practitioner (AP) in today’s clinical arena. Society members will include advanced practice professionals specializing in oncology, including nurse practitioners (NPs), physician assistants (PAs), clinical nurse specialists, pharmacists, and others.

The new Society was launched at the JADPRO Live meeting held this past January in St. Petersburg, Florida. Over 150 of the registered attendees signed up to become charter members of the new Society. The Society’s goals are to improve the quality of care for patients by supporting critical issues in the educational, clinical, and professional development of APs in oncology. Collaboration among all oncology providers is a critical focus of the Society as it supports the continued improvement in quality of care.

**Figure 1:**
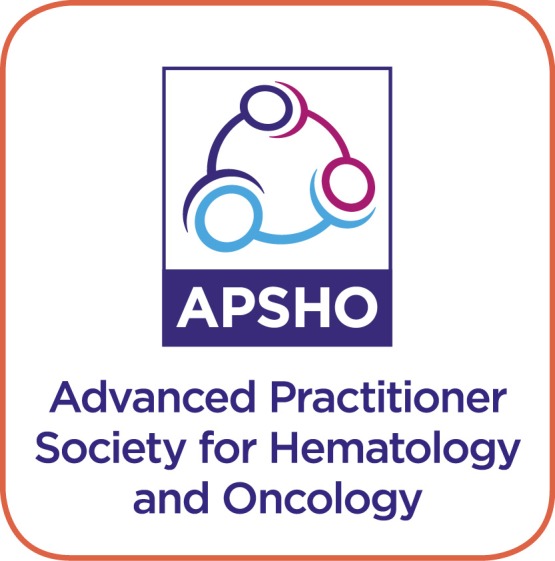
APSHO

As we experience ongoing changes in health care, the role of the AP continues to expand. The ASCO Study of Collaborative Practice Arrangements (Towle, 2011) demonstrated that the extension of oncology services with collaborative practice models using NPs and PAs is not only feasible but also an essential part of the health-care environment of today and tomorrow. The study showed that both professionals and patients experienced satisfaction with the care given by APs; additionally, an increase in productivity was noted. 

The new Society will provide a forum for the oncology AP, with focused, relevant educational opportunities for continued growth. Here are some of the proposed benefits members of APSHO will receive:

- Reduced registration fees for future APSHO conferences, including JADPRO Live
- Opportunity to earn free CME/CE credits
- Complimentary subscription to JADPRO
- Subscriptions to a quarterly APSHO newsletter, JNCCN, and The ASCO Post
- Opportunity to serve on APSHO committees and focus groups

As your role as an oncology AP continues to expand, your educational, legislative, and clinical needs will grow as well. APSHO promises to be a unique society that is focused on your specialized needs. But your participation is essential! Please visit our webpage at www.apsho.org and register to receive information and updates about this exciting new endeavor. We look forward to collaborating with you.
